# Postpartum depression and the moderating role of empathy on child physiological reactivity

**DOI:** 10.1186/s13052-025-02063-y

**Published:** 2025-07-15

**Authors:** Elisa Cainelli, Barbara Carretti, Sara Puddu, Filippo Zemin, Paola Veronese, Damiano Menin, Marco Dondi, Agnese Suppiej

**Affiliations:** 1 Department of General Psychology, University of Padova, Padova, Italy; 2 Gynecology and Obstetrics Unit, Department of Women’s and Children’s Health, University Hospital of Padova, Padova, Italy; 3 Department of Human Studies, University of Ferrara, Ferrara, Italy; 4 Department of Medical Science, University of Ferrara, Ferrara, Italy

**Keywords:** Prenatal, Postnatal, Depression, Empathy, Heart rate variability, Attachment, Mother-child bond

## Abstract

**Background:**

Perinatal maternal depression is a risk factor for the development of psychopathology in the offspring, probably by impacting its early physiological reactivity to stress and emotion regulation. The objective of this study is to evaluate the influence of postpartum depressive symptoms on the offspring’s physiological reactivity (heart rate variability -HRV) and the possible moderating effects of empathy and perinatal attachment.

**Methods:**

We recruited 24 mother-child dyads in a prospective observational study. The psychopathological profile and personal characteristics of pregnant women at 24–28 gestational weeks were evaluated by administrating the Perinatal Attachment Inventory (PAI) and the Empathy Quotient (EQ) questionnaire. Three weeks after childbirth, women completed the Edinburgh Postnatal Depression Scale (EPDS), and the offspring underwent an HRV recording.

**Results:**

Postpartum depression was a good predictor of the offspring’s HRV activity (b = 1.49, *p* =.045). Both perinatal attachment (*r*=-.463, *p* =.023, Fisher’s ES=-0.501) and empathy (*r*=-.570, *p* =.004, Fisher’s ES=-0.648) were negatively associated with postpartum depression. Finally, empathy showed a moderator effect on the association between postpartum depression and child’s HRV (b = 0.13, *p* =.03). We found no moderator effects for Perinatal Attachment.

**Conclusion:**

Despite the preliminary nature of our data, maternal depression showed predictive power on offspring’s early regulatory mechanisms, with possible knock-on effects on emotional regulation and mother-child bond establishment. Interestingly, some maternal personality characteristics, such as empathy, can influence the development of depression symptoms, suggesting the presence of moderator factors modulating the association between maternal psychological status and child physiological reactivity.

**Supplementary Information:**

The online version contains supplementary material available at 10.1186/s13052-025-02063-y.

## Introduction

Maternal perinatal depression has detrimental effects on child development. Specifically, it has been associated with an increased risk of a wide range of adverse outcomes, including cognitive, social, and emotional disturbances, depression and anxiety, behavioral problems, and attachment insecurity [1–6]. This is particularly relevant given the high prevalence of depression during the perinatal period, with approximately 18% of women experiencing depressive symptoms at some point during pregnancy (7–13% per trimester) [7, 8]. Among women without a prior history of psychopathology, the overall prevalence of postpartum depression is approximately 17% [9]. The prevalence of perinatal depression risk in Italy is similar to that reported in other countries, with a pooled prevalence of 20.2% prepartum and 11.1% postpartum [10].

The mechanisms underlying the association between maternal depression and adverse infant and child developmental outcomes have been extensively studied [6, 11], with particular attention to how depression interferes with mother-child bonding [12–17]. It has been documented that mothers with depression engage in less intense interactions with their children, experience higher levels of stress, and perceive their children more negatively [18]. This pattern of mother-child interaction has also been observed among mothers exhibiting subclinical levels of depressive symptoms [19–21].

As a consequence of maternal depression, newborns often display more dysregulated behaviors, such as disturbed or disorganized sleep and difficult temperament [22, 23], which may, in turn, exacerbate negative symptoms in the mother, creating a vicious cycle [24]. However, certain maternal characteristics can mediate or moderate the mother–child relationship. For instance, women with a disorganized attachment style appear particularly vulnerable [25]. Conversely, maternal sensitivity—the ability to accurately perceive and interpret infant signals and respond contingently and appropriately—promotes healthy mother–child interactions [26] and aids in regulating the infant’s stress responses and emotional regulation, fostering long-term wellbeing [27–29].

In response to poor maternal interaction, infants may alter their behavior, exhibiting poorer emotional and behavioral regulation, fewer positive and more negative facial expressions, avoidance, and increased fussiness [30, 31]. Alterations in newborn behavioral regulation can be objectively measured via heart rate variability (HRV), a reliable indicator of autonomic nervous system (ANS) function, particularly the vagal branch, and thus an index of regulatory capacity and environmental adaptation [32, 33]. The parasympathetic (vagal) component of HRV, reflected in the high-frequency (HF) band, is a candidate biomarker of physiological stress regulation through top-down cognitive control across the lifespan [34–36]. Supporting this, previous studies have reported that HRV in the prenatal and neonatal periods predicts various developmental outcomes, including language skills and psychological development in early childhood [37–39]. Furthermore, a prior study demonstrated that maternal HRV influences infant neurophysiology via alterations in autonomic stress regulation and dyadic physiological co-regulation, highlighting the impact of maternal mental health on child brain development [40].

To our knowledge, no studies have investigated the effects of postpartum depressive symptoms on neonatal HRV. We focused specifically on the postnatal period—a time of significant psychological vulnerability for the mother due to neurocognitive and neuroendocrine changes. These changes are supported by transient high brain plasticity [41] which sustains maternal behaviors focused on the child, ensuring their central role in neuroplastic adaptations [41–43]. While these brain changes facilitate maternal adaptation to the child’s needs and development, they also increase vulnerability to psychopathology [43]. Hence, the early postpartum months are critical because the same neurobiological modifications that enable maternal caregiving and bonding also predispose the mother to psychopathological risk [44].

Based on existing literature, the first aim of this study was to examine the relationship between maternal postpartum depression and the regulatory abilities of their infants, as indexed by the HF component of HRV. The second aim was to assess the moderating role of protective factors, specifically maternal empathy and perinatal attachment, on this relationship. Consistent with prior findings, we hypothesized that maternal postpartum depressive symptoms would be associated with alterations in the developing child’s psychophysiological stress response and regulatory capacities (Aim 1). Previous research, mainly in adults and older children, has focused on the HF component of HRV, typically finding reductions associated with various distress conditions, including depression [45, 46], although some studies have questioned the reliability of HF as a biomarker [47]. In neonates, this relationship is more complex, as the parasympathetic system undergoes rapid development in the first weeks after birth, marking this period as particularly sensitive and vulnerable [48, 49]. Despite limited research, decreases in HF have generally been reported following stress exposure in neonates [48]. Therefore, we hypothesized that maternal postpartum depression would reduce the infant’s parasympathetic activity (HF), reflecting a dysmaturity of the ANS.

Regarding the second aim (Aim 2), we hypothesized that maternal characteristics such as empathy and perinatal attachment would be associated with the development of postpartum depressive symptoms and could moderate or mitigate the impact of maternal depression on infant HRV.

## Methods

### Participants

After excluding dyads with incomplete data, the study included 24 mother–child dyads recruited during pregnancy between March 2022 and February 2023 at the Gynecology and Obstetrics Unit of the Department of Women’s and Children’s Health, University Hospital of Padova. The sample size was limited due to practical constraints related to the specific clinical setting and inherent recruitment challenges. However, this sample size is comparable to that of previous similar studies [50, 51]. The inclusion and exclusion criteria are reported for clarity in Table 1.

**Table 1:** The table summarizes the inclusion and exclusion criteria of the participants

**Inclusion criteria (mother)**	First evaluation at a gestational period 24–28
	Informed consent
**Inclusion criteria (child)**	At term birth
	Normal fetal medical records
**Exclusion criteria (mother)**	Age < 18 years
	Illnesses complicating pregnancy and delivery
	Use of corticosteroid medication
	Incomplete medical and demographic records
**Exclusion criteria (child)**	Illnesses complicating delivery and perinatal period
	Incomplete medical records

The women recruited had a median age of 32 years (31; 36.7), and they were evaluated in the prenatal period at a median gestational age of 28 weeks (25.25; 29). None of the mothers assumed alcohol or smoked during pregnancy, and none had a psychiatric or neurological diagnosis; the socioeconomic status was medium for 20/24 women, low for 3/24, and high for 1/24 according to [52]. Complete data on demographic and clinical characteristics of the women are shown in the Supplementary Material.

Children were born at a median gestational age of 38.5 weeks (38; 41), median birth weight of 2996 g (2670; 3697); 14/24 were male. None of the children had perinatal complications or needed resuscitation. As for mothers, complete data on children’s demographic and clinical characteristics are shown in the Supplementary Material.

The study received approval from the Institutional Review Board (Prot.n 24139, 02.2022 Comitato Etico per la Sperimentazione Clinica dell’Azienda Ospedaliera di Padova, and 193-c, 01.2024 Comitato Etico della Ricerca Psicologica Area 17). It was conducted in accordance with ethical guidelines.

### Material

#### Psychological measures in the mother

*Empathy Quotient (EQ)* [53] The EQ is a self-report questionnaire that measures empathy’s cognitive and affective aspects. The Italian version is a 60-item questionnaire with 40 questions regarding empathy and 20 filler items. The final score ranges between 0 and 80, with higher scores implying higher empathy. A cut-off < 30 has been demonstrated to differentiate autism spectrum conditions from control participants [53, 54]; no other cut-offs are available. The Italian version of the EQ has good validity and excellent reliability [53]; an acceptable replication of the original three-factor solution has been provided, yielding the three subscales (cognitive empathy, emotional reactivity, and social skills) with high internal and test-retest reliability [53]. Specifically, the reliability analysis of the questionnaires yielded Cronbach’s alpha values of 0.86 in the current sample and 0.79 in the standardization sample.

*The Prenatal Attachment Inventory (PAI)* [55] is a questionnaire that measures prenatal attachment in five dimensions: fantasy, affection, interaction, sensitivity, and differentiation of self from the fetus. It is composed of 21 Likert-type items, and the total score is the sum of the items and ranges from 21 to 84, with higher scores indicating higher levels of prenatal attachment. No cut-offs are available. When used as a global score, the PAI has been shown to provide adequate reliability and validity in Italian women [55]. Specifically, the reliability analysis of the questionnaires yielded Cronbach’s alpha values of 0.88 in the current sample and 0.87 in the standardization sample.

*The Edinburgh Postnatal Depression Scale (EPDS)* [56, 57]. The EPDS is a 10-item self-report scale to screen for Postnatal Depression. Each item has four answer choices indicating different levels of severity (0–3 points). Each item scores between 0 and 3, totaling 0 to 30 points overall. Higher scores denote more significant symptoms of postnatal depression. We chose the 9/10 cut-off score for the use of the scale in community surveys and screening [56, 57]. The evaluation of the validity of EPDS in the Italian version provided good results [56]; the effectiveness of the range of the selected cut-off scores was also confirmed [56, 57]. Specifically, the reliability analysis of the questionnaires yielded Cronbach’s alpha values of 0.89 in the current sample and 0.79 in the standardization sample.

#### Autonomic measures– HRV in the child

Autonomic functioning has been estimated by extracting HRV from an electrocardiogram (ECG). Ag/AgCl surface electrodes were positioned on the chest in a modified lead II configuration. The raw ECG signal was recorded with a LiveAmp system (Brain Products, Gilching, Germany) and amplified with a gain of 150, bandpass filtered (0.3–100 Hz), and digitized at 500 Hz (16 bit A/D converter, resolution 0.559 µV/LSB). The raw ECG signal was then exported to Kubios HRV Analysis Software 2.2 (The Biomedical Signal Analysis Group, Department of Applied Physics, University of Kuopio, Finland) to estimate the occurrence of each heartbeat and derive the series of inter-beat intervals (IBIs), calculated as the difference in milliseconds (ms) between two consecutive R-waves. Fast Fourier spectral analysis was conducted on the IBI series to compute frequency domain indexes and obtain the HF power (0.15–0.40 Hz) component. HF Frequency-domain index was logarithmically transformed to normalize its distribution.

### Procedure and study design

This is a longitudinal prospective observational study, organized in two moments: prenatally and postpartum. After the recruitment, at 24th -28th gestational weeks, the pregnant women underwent a structured interview about clinical and demographic information (complete data obtained are shown in the Supplementary Material) and filled out the Empathy Quotient (EQ) questionnaire and the Perinatal Attachment Inventory (PAI). The mothers were asked to return to our department with their sons once the baby was born between the third and fourth weeks of life. All appointments took place at our Department between 9 and 10 a.m. As the dyads came, children were prepared for the HRV recording by placing him/her on a crib and being undressed just enough to place the two electrodes. After the procedure, if the child became nervous, he/she was calmed down by the mother until the child reached a drowsy state and the recording could begin. The electrocardiogram recordings have been recorded for sufficient time to obtain at least five minutes artifact-free. After the recordings, the mothers filled out additional questionnaires about the possible presence of postnatal depression, the Edinburgh Postnatal Depression Scale (EPDS). A structured interview has also collected information about delivery and the perinatal period. Questionnaires were administered using a computer interface and secure REDCap link (https://www.projectredcap.org).

### Statistical analysis

At first, correlations between prenatal attachment (PAI), empathy (EQ), postpartum depression (EPDS) scores, and HF component of the child’s HRV were run using Pearson r. False discovery rate correction for multiple comparisons has been made. Independent linear regressions were conducted to evaluate the predictive power of postpartum depression on the HF component of the child’s HRV. Subsequently, the moderator effects of perinatal attachment and empathy were evaluated by adding them to the regressions and evaluating the interactions between perinatal attachment and postpartum depression in one model, empathy and postpartum depression in the other, on the HF. Cronbach’s alpha measure of reliability has been performed to evaluate consistency between items.

Statistics were determined using JASP (The JASP team 2018, version 0.19.2).

## Results

### Descriptive statistics

Table 2 reports the scores obtained by the women in the questionnaires.

**Table 2:** Median, 25- 75th percentiles, and number of clinical scores (where applicable) obtained by the mothers at questionnaires in the postpartum (EPDS) and in the prenatal period (EQ, PAI) (*n* = 24)

	PRENATAL		POSTPARTUM
	Empathy (EQ)	Perinatal Attachment (PAI)	Depression (EPDS)
Median	43.0	-0.23	5.5
25th percentile	38.0	-0.60	2.5
75th percentile	53.0	0.77	10
N clinical scores	--	--	9/24

Legend: EPDS: Edinburgh Postnatal Depression Scale; EQ: Empathy Quotient; PAI: Perinatal Attachment Inventory

The median HF percentage values in children were 8.200 Hz (3.425; 10.275), while the days of life at the recordings were 25 (20; 30 days). HF Frequency-domain values after logarithmical normalization are reported in Table 3.

**Table 3:** HRV values in the HF Frequency-domain after logarithmical normalization

	HF
Median	2.025
25th percentile	1.163
75th percentile	2.297
Mean	1.790
SD	0.893

### Correlations

**Fig. 1:**
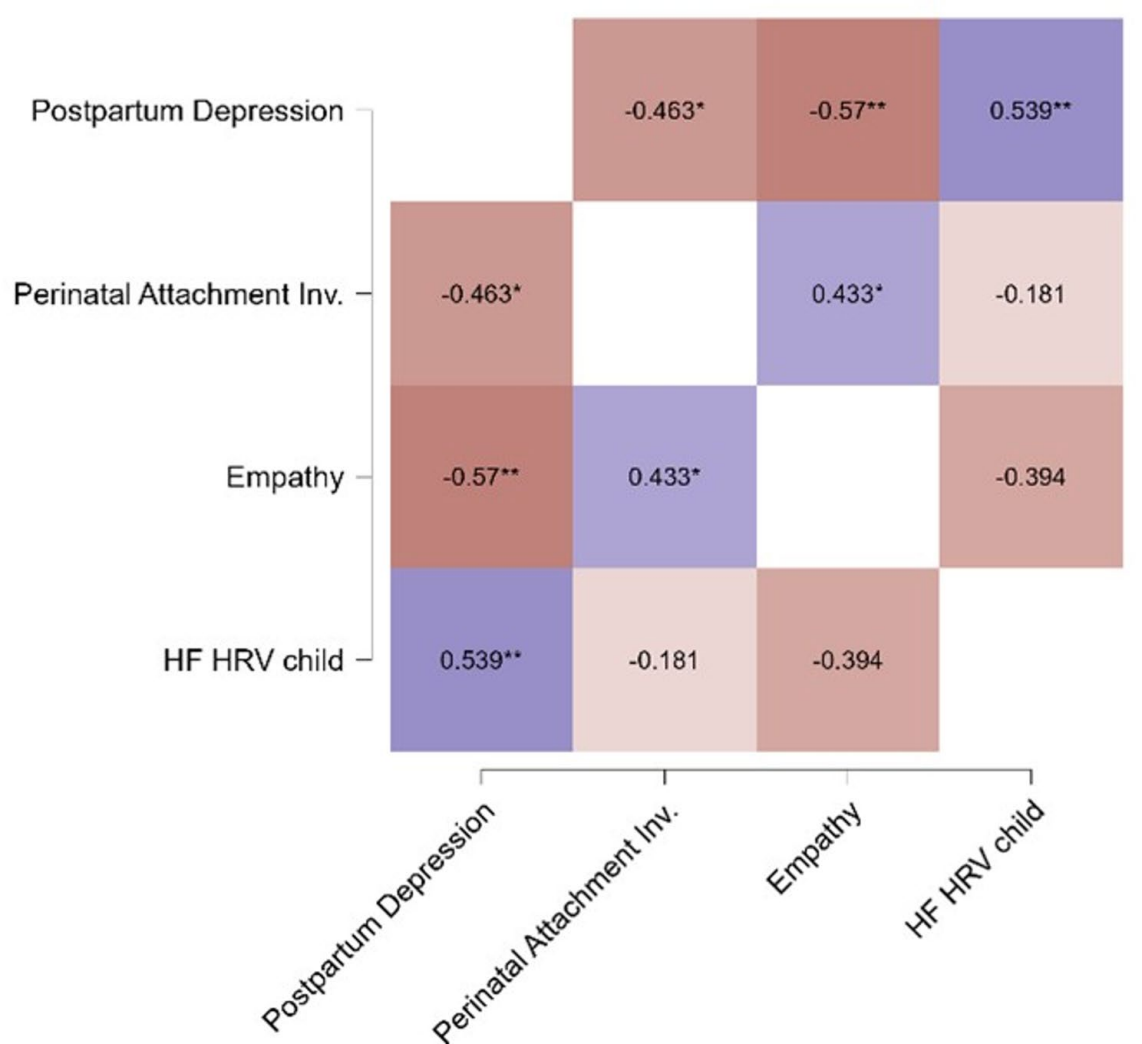
Pearson r heatmap represents the associations between postpartum depression, perinatal attachment, empathy, and HF component of the child’s HRV. HF: high frequency

Correlations between the measures of interest were run (see Fig. 1). The HF scores of the child’s HRV were significantly correlated only with the postpartum depression score obtained in the Edinburgh Postnatal Depression Scale (*r* =.539, *p* =.007, Fisher’s ES = 0.603). Postpartum depression was inversely correlated both with the Empathy Quotient (*r*=-.570, *p* =.004, Fisher’s ES=-0.648) and Perinatal Attachment Inventory (*r*=-.463, *p* =.023, Fisher’s ES=-0.501). The correlations remained significant after correction for the false discovery rate.

### Predictors of neonate’s HF component

At first, a linear regression was run to examine the contribution of postpartum depressive symptoms to the neonate’s HF component. Postpartum depressive symptoms significantly predicted the neonate’s HF component (b = 1.59, st. error = 0.53, 95% C.I. 0.49, 2.69; *p* =.007).

**Fig. 2:**
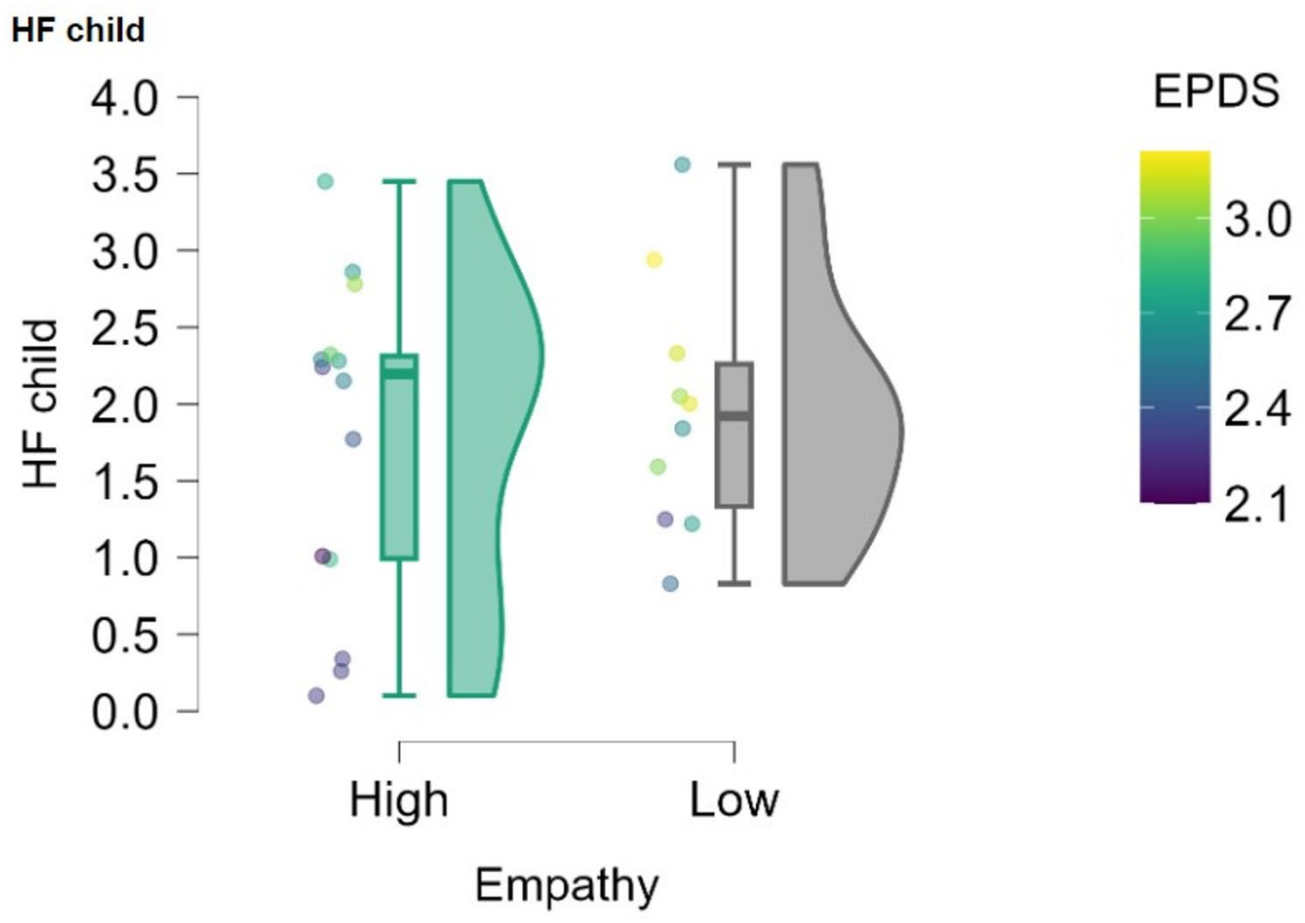
The figure represents the rainclouds of the distribution of HF values in women with high and low empathy, also highlighting the postpartum depression (EPDS) values reported

By adding in the regressions EQ and PAI, a moderation effect emerges between the Empathy Quotient and Postpartum Depression (b = 0.13, st. error = 0.05, 95% C.I. 0.014, 0.24; *p* =.03), while the principal effect of Postpartum Depression alone is no longer significant: higher levels of empathy predict lower symptoms of postpartum depression and stress, which in turn are associated with lower HF values (Fig. 2), suggesting that the association between variables is more complex than a simple principal effect. Therefore, empathy is shown to moderate the effect of depression and stress on the child’s HRV by reducing them (Fig. 2). We did not find moderation effects for Perinatal Attachment.

## Discussion

The first aim of our study was to investigate the effects of postpartum depressive symptoms on the offspring’s HF component (parasympathetic) of HRV, which, as a measure of stress responsiveness and regulatory capacity, may represent a key mechanism in the establishment of psychological vulnerability (Aim 1). We also aimed to explore the associations of maternal empathy and perinatal attachment with postpartum depressive symptoms and their potential moderating effects on offspring HRV (Aim 2).

Regarding Aim 1, we found that the development of postpartum depressive symptoms is significantly associated with an increase in the offspring’s HF component. This association suggests that the mother’s psychological state, likely through its influence on mother-child interaction, impacts the early regulatory abilities of the infant, as reflected by the HF component of HRV, a well-established indicator of parasympathetic ANS activity. By considering the effects on the offspring’s HRV of depression associated with pregnancy, we found a similar study in the literature, but evaluating the effect of prenatal depression on child’s autonomic measures [58]. In this work by Jacob et al. (2009), the authors found that total HRV was significantly lower in children of mothers with a history of major depressive disorder and life stressors [58]. Other investigations have focused on maternal psychopathology during pregnancy, often in the context of “prenatal stress-immune programming” [49, 59–63]. Since the women in our study had no prior diagnosis of depression or other psychopathologies, to our knowledge, this is the first study to evaluate the effects of postpartum depression on the early regulatory capacities of the child as indexed by HRV.

Contrary to our initial hypothesis, postpartum depression was associated with an increase—not a decrease—of the offspring’s HF component. Typically, fetal stress is associated with a reduction in parasympathetic (HF) activity; however, increases have also been documented. For example, perinatal asphyxia can cause bradycardia with a consequent rise in parasympathetic tone [34, 64]. Similar increases have been observed in infants prenatally exposed to opiates and in premature infants who developed apparent life-threatening events post-discharge from neonatal intensive care [65, 66]. The understanding of the impact of maternal depression on healthy, full-term newborns—particularly during the critical period of rapid parasympathetic maturation reflected by HF—is limited. The ANS may respond differently depending on the maturation stage and nature of the stressor [49], rendering it vulnerable to perinatal influences [67, 68]. Early adversities can significantly impair ANS responsiveness to environmental challenges, with consequent effects on neurodevelopment [67, 69–71]. However, from an evolutionary point of view, it has also been hypothesized that stressful conditions at critical periods of development might induce an increased susceptibility to the surrounding environment that might not necessarily be maladaptive and might have the effect of faster maturation [72]. Interestingly, our previous work on the long-term outcome after at-term asphyxia revealed a significant resting-state increase in HF, interpreted as a compensatory parasympathetic predominance [68].

It is important to note that HRV is an estimate of autonomic function and reflects complex, potentially nonlinear control mechanisms. HF, in particular, results from the intricate integration of respiratory and cardiac activity. The role of respiration in HRV interpretation remains a debated topic [47, 73, 74], especially in neonates, where respiratory control is still maturing and represents a key developmental milestone post-birth [75]. A measure of respiration would have greatly aided the interpretation of our HF findings, representing a notable limitation of this study. Thus, caution is warranted when interpreting the significance of increased HF under our experimental conditions.

After perinatal depression, not all infants exposed to maternal depression will exhibit negative outcomes [76, 77]. The relationship between maternal depression, maternal behavior, and child outcomes is likely complex and moderated by various factors [76]. Depression and its characteristics (e.g., severity, persistence) can be associated with personality traits and personal characteristics, such as empathy [78]. Therefore, we were interested in exploring if some maternal characteristics could moderate the development of psychopathological symptomatology and moderate the effects of depression on the child’s HRV (Aim 2). We found that prenatal attachment—a construct encompassing maternal sensitivity, fantasy, interaction, affection, and differentiation of self from the fetus—negatively correlated with postpartum depressive symptoms, suggesting that positive prenatal attachment may confer protective effects extending into the postnatal period. Prior studies have identified prenatal maternal attachment as a key factor in promoting maternal-child bonding, facilitating better mother-child interactions, and enhancing maternal self-efficacy, thereby reducing the risk of postpartum depression [79, 80].

However, perinatal attachment was not associated with infant HRV and did not moderate the relationship between depressive symptoms and HRV. In contrast, maternal empathy was negatively associated with depressive symptoms and significantly moderated the effects of depression on infant HRV. Specifically, higher maternal empathy was linked to lower depressive symptoms and correspondingly reduced HF power in offspring.

Since sensitive parenting requires an accurate and empathic response to infant cues, empathy may be a crucial mechanism underlying maternal sensitivity. We hypothesize that the ability to imagine and empathize with the infant facilitates better emotion regulation. Neurobiologically, brain plasticity mechanisms occurring in postpartum women involve circuits associated with empathy [81]. Supporting this, previous studies have found correlations between empathy, reduced depressive symptoms, and less remote maternal behavior in the early postpartum period; higher empathy scores were associated with more engaged and less depressive maternal behavior [82–84].

This aspect opens important scenarios for the prevention of postnatal depressive symptoms, with cascading effects on the relationship with the child first and on the child’s neurodevelopment later. If further research confirms empathy’s role in mitigating postpartum depression and fostering mother–child bonding, intervention programs enhancing empathy in pregnant women could better prepare them for the demands of early motherhood. Mindfulness- and compassion-based interventions—which are closely linked to empathy—have shown effectiveness in reducing depression symptoms during pregnancy and postpartum [85, 86].

Beyond the already mentioned, this study has several limitations. The most significant is the small sample size, which limits statistical power and the generalizability of our findings. The small sample increases the risk of Type II errors, potentially obscuring subthreshold effects, such as the moderating role of perinatal attachment. Nonetheless, the observed effect sizes and exploratory nature of the study justify the current sample size as a pragmatic starting point. Thus, our findings should be considered preliminary, and replication with larger samples is necessary to confirm these results with greater statistical rigor.

Additionally, the correlational design precludes causal inference and limits understanding of developmental trajectories. Longitudinal follow-up is needed to clarify the clinical significance of HRV differences observed. Finally, our participants did not have clinical diagnoses of postpartum depression but exhibited varying symptom severity, allowing only correlational analysis rather than case-control comparisons. The effect of potential variables (e.g., breastfeeding, sleep deprivation, partner support) was not evaluated, even given the small sample size that would not have allowed for statistical control of them.

## Conclusions

In conclusion, we found that postnatal depression is associated with the autonomic functioning of the offspring, suggesting that maternal psychopathology may interfere with the child’s early regulatory mechanisms, with possible knock-on effects on the responses to stress, mother-child bond, and emotional regulation later in development. However, some maternal personal characteristics, such as perinatal attachment and empathy, can influence the development of depressive symptoms, suggesting possible moderators/modulating factors in the association of maternal depression and child autonomic changes. A better understanding of how maternal characteristics may affect the development of psychopathology in the child may open up interesting possibilities for early intervention.

## Electronic supplementary material

Below is the link to the electronic supplementary material.

Supplementary Material 1

## Data Availability

Data will be available on request.

## Abbreviations

ANS: Autonomous nervous system
EPDS: Edinburgh Postnatal Depression Scale
EQ: Empathy quotient
HF: High frequency
HRV: Heart rate variability
PAI: Parental Attachment Inventory
