# Supplementary material for: Postpartum depression and the moderating role of empathy on child physiological reactivity
Source: Ital J Pediatr. 2025 Jul 15;51:223. doi: 10.1186/s13052-025-02063-y (PMC12261772; doi:10.1186/s13052-025-02063-y)
Supplement: Supplementary file 1 — Supplementary Material 1 [file 13052_2025_2063_MOESM1_ESM.docx]

Table 1. Demographic and clinical characteristics of the maternal sample

|  | Median (Q1; Q3), N/tot |
| --- | --- |
| Age, y | 32 (31; 36.7) |
| Weight (pre-pregnancy), kg | 73 (55; 83.5) |
| Weight gain, kg | 3 (-2; 8) |
| Foreign | 1/24 |
| Gestational age in the prenatal period, weeks | 28 (25.25; 29) |
| Gemellarity | 2/24 |
| Actually Working | 11/24 |
| Education > 12 years | 24/24 |
| Smoke | 0/24 |
| Alcohol | 0/24 |
| Sport | 8/24 |
| Medication |  |
| SES |  |
| Low | 3/24 |
| Medium | 20/24 |
| High | 1/24 |

Legend: SES: socio-economic status

The clinical characteristics of the offspring and of childbirth are reported in Table 2.

Table 2. Clinical characteristics of the offspring and of childbirth.

|  | Median (Q1; Q3), N/tot |
| --- | --- |
| Gestational age, weeks | 38.5 (38; 41) |
| Days of life at recording | 25 (20; 30) |
| Male: female rate | 14:12 |
| Apgar 1°minute | 9 (9; 9) |
| Apgar 5° minutes | 10 (10; 10) |
| Apgar 10° minutes | 10 (10; 10) |
| Reanimation | 0/24 |
| Birth weight, gr | 2996 (2670; 3697) |
| Birth length, cm | 49 (47.25; 50) |
| Cranial circumference, cm | 34.5 (33.1; 35) |
| Apneas | 1/24 |
| Infections in the neonatal period | 1/24 |
| Type of delivery |  |
| Vaginal | 14/24 |
| Cesarean | 3/24 |
| Induction | 7/24 |
| Delivery complications | 0/24 |
